# Diversity of Secondary Metabolism in Aspergillus nidulans Clinical Isolates

**DOI:** 10.1128/mSphere.00156-20

**Published:** 2020-04-08

**Authors:** M. T. Drott, R. W. Bastos, A. Rokas, L. N. A. Ries, T. Gabaldón, G. H. Goldman, N. P. Keller, C. Greco

**Affiliations:** a Department of Medical Microbiology and Immunology, University of Wisconsin—Madison, Madison, Wisconsin, USA; b Department of Bacteriology, University of Wisconsin—Madison, Madison, Wisconsin, USA; c Faculdade de Ciências Farmacêuticas de Ribeirão Preto, Universidade de São Paulo, São Paulo, Brazil; d Department of Biological Sciences, Vanderbilt University, Nashville, Tennessee, USA; e Faculdade de Medicina de Ribeirão Preto, Universidade de São Paulo, Ribeirão Preto, Brazil; f Life Sciences Program, Barcelona Supercomputing Centre, Barcelona, Spain; g Mechanisms of Disease Program, Institute for Research in Biomedicine, Barcelona, Spain; h ICREA, Barcelona, Spain; University of Georgia

**Keywords:** *Aspergillus nidulans*, secondary metabolism, intraspecific variation, horizontal gene transfer, viridicatumtoxin

## Abstract

The filamentous fungus Aspergillus nidulans has been a primary workhorse used to understand fungal genetics. Much of this work has focused on elucidating the genetics of biosynthetic gene clusters (BGCs) and the secondary metabolites (SMs) they produce. SMs are both niche defining in fungi and of great economic importance to humans. Despite the focus on A. nidulans, very little is known about the natural diversity in secondary metabolism within this species. We determined the BGC content and looked for evolutionary patterns in BGCs from whole-genome sequences of two clinical isolates and the A4 reference genome of A. nidulans. Differences in BGC content were used to explain SM profiles determined using liquid chromatography–high-resolution mass spectrometry. We found that in addition to genetic variation of BGCs contained by all isolates, nine BGCs varied by presence/absence. We discovered the viridicatumtoxin BGC in A. nidulans and suggest that this BGC has undergone a horizontal gene transfer from the *Aspergillus* section *Nigri* lineage into *Penicillium* sometime after the sections *Nigri* and *Nidulantes* diverged. We identified the production of viridicatumtoxin and several other compounds previously not known to be produced by A. nidulans. One isolate showed a lack of sterigmatocystin production even though it contained an apparently intact sterigmatocystin BGC, raising questions about other genes and processes known to regulate this BGC. Altogether, our work uncovers a large degree of intraspecies diversity in BGC and SM production in this genetic model species and offers new avenues to understand the evolution and regulation of secondary metabolism.

**IMPORTANCE** Much of what we know about the genetics underlying secondary metabolite (SM) production and the function of SMs in the model fungus Aspergillus nidulans comes from a single reference genome. A growing body of research indicates the importance of biosynthetic gene cluster (BGC) and SM diversity within a species. However, there is no information about the natural diversity of secondary metabolism in A. nidulans. We discovered six novel clusters that contribute to the considerable variation in both BGC content and SM production within A. nidulans. We characterize a diverse set of mutations and emphasize how findings of single nucleotide polymorphisms (SNPs), deletions, and differences in evolutionary history encompass much of the variation observed in nonmodel systems. Our results emphasize that A. nidulans may also be a strong model to use within-species diversity to elucidate regulatory cross talk, fungal ecology, and drug discovery systems.

## Abstract (precis)

Much of what we know about the genetics underlying secondary metabolite (SM) production and the function of SMs in the model fungus Aspergillus nidulans comes from a single reference genome. A growing body of research indicates the importance of biosynthetic gene cluster (BGC) and SM diversity within a species. However, there is no information about the natural diversity of secondary metabolism in A. nidulans. We discovered six novel clusters that contribute to the considerable variation in both BGC content and SM production within A. nidulans. We characterize a diverse set of mutations and emphasize how findings of single nucleotide polymorphisms (SNPs), deletions, and differences in evolutionary history encompass much of the variation observed in nonmodel systems. Our results emphasize that A. nidulans may also be a strong model to use within-species diversity to elucidate regulatory cross talk, fungal ecology, and drug discovery systems.

## INTRODUCTION

Secondary metabolites (SMs) are small molecules that, by definition, are not required for primary growth of the organisms that produce them but, instead, are associated with specific lifestyles of many fungi (1), including host range expansions of several fungal pathogens (2–5). SMs may also provide fitness benefits to the fungi that produce them under specific nutrient environments (6) and ecological conditions (7, 8). In addition to having a defining role in fungal lifestyle, many SMs produced by fungi are also of enormous economic value to humans (e.g., penicillin and lovastatin) (9). The A4 reference isolate of Aspergillus nidulans has been the main model system used to study the genetics and regulation of SM production by biosynthetic gene clusters (BGCs) (10–13). This work has enabled the development of genomic scans that identify BGCs in genetic sequence data, a leading approach used to understand both the ecology of fungi and to harness these bioactive molecules for human use (14, 15). However, both ecological and applied inferences from such scans are complicated by the fact that the presence of a BGC in a fungal genome is not always predictive of an SM being produced. Part of this discrepancy is thought to arise from within-species diversity as production of SMs can vary substantially between isolates of the same species (16, 17). While work in A. nidulans has proven to be important for the discovery and manipulation of genes associated with secondary metabolism in fungi, the use of this model system to better understand how intraspecific variation affects SM production has been precluded by an exclusive focus on a single reference isolate that provides no context of the diversity found in this species.

The focus on a single isolate of A. nidulans partially reflects that efforts to understand the diversity of secondary metabolism in fungi has focused on differences between species. While BGCs typically exhibit narrow taxonomic distributions (18, 19) with extensive overlap between closely related species, there can also be considerable variation between species (20). Even when BGC contents are highly similar between closely related species, SM production may be quite differentiated (21). However, it is not clear if such differences are fixed in these species, or whether the individual isolates used for these comparisons do not fully represent the secondary metabolome of their species. Such differentiations have been made difficult by a focus on the presence or absence of functional alleles of a small number of important toxins (22, 23). This focus on a select subset of BGCs in some species means that variation in dozens of other BGCs present in these species has largely gone unrecognized. Recently Lind et al. (24) observed that there is considerable variation between the genomes of Aspergillus fumigatus isolates both in the genetic similarity and in the total count of BGCs. The genomes of some isolates were missing or had pseudogenized variants of up to ∼30% of BGCs. Our increasing understanding of the intraspecific variation in BGC content indicates an important and largely unexplored source for new drug discovery (14) but also raises questions of whether some inferences of fungal secondary metabolism represent a characteristic of a single isolate rather than a species-level pattern.

While many of the fundamental insights into fungal secondary metabolism resulting from the A. nidulans model system have been substantiated across species and genera, there has been little effort to use this system to help contextualize our understanding of intraspecific variation in secondary metabolism. In a rare example looking beyond the A4 isolate, Bastos et al. (25) recently characterized several aspects of genomic and phenotypic variation of two A. nidulans clinical isolates. Even with this small sample, they found that isolates varied significantly from the A4 reference strain in primary growth, sexual development, and resistance to oxidative stress. While the authors did not comprehensively assess differences in secondary metabolism, the aforementioned traits have been associated with the production of, or inability to produce, specific SMs. For example, in A. nidulans decreased production of sterigmatocystin (ST) is associated with decreased sporulation (26), decreased production of sexual spores (27), and decreased resistance to oxidative stress (28–30). The diversity found by Bastos et al. (25) raises the question of whether previous associations observed in A4 are recapitulated in other isolates of this species. In addition to associations of secondary metabolism and other fungal traits, BGCs in A. nidulans can have important regulatory cross-talk interactions that can result in the production of SMs from otherwise silent clusters (31). However, without understanding which BGCs are common to isolates of A. nidulans and if A4 has all of these clusters, it is not possible to elucidate the extent of such cross talk interactions. Uncovering the diversity in secondary metabolism that is natural within A. nidulans will add to a growing understanding of the diversity in BGCs within fungal species and will also contextualize existing work on fungal genetics, ecology, and secondary metabolism evolution, thus providing new areas of future research.

In this study, we sought to determine if there is variation in BGC content and SM production of two clinical A. nidulans isolates in comparison to these features of the worldwide workhorse isolate A4 (32). Additionally, we characterize the types of mutations that have led to variation in these traits. We interpret this diversity in the context of phenotypic differences observed by Bastos et al. (25) to suggest areas of future research. Specifically, we addressed the following questions: (i) Is there variation in BGC content and SM production within A. nidulans? (ii) What mutations affect BGC content and SM production in A. nidulans?

## RESULTS

### Variation in BGC count.

**FIG 1:**
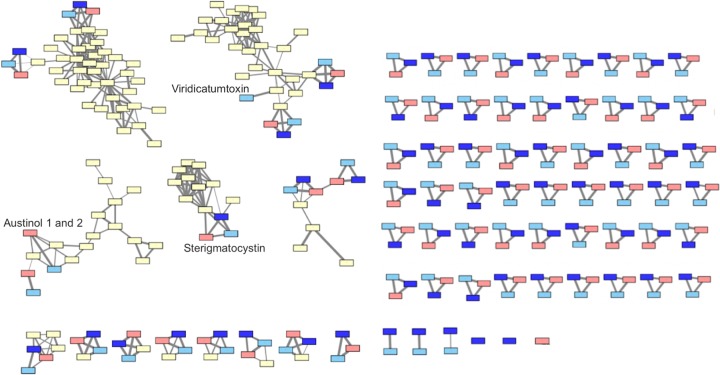
Network of biosynthetic gene clusters (BGCs) from Aspergillus nidulans. Boxes represent the A4 reference isolate (red), the two clinical isolates MO80069 (light blue) and SP260548 (dark blue), and clusters from the MIBiG repository of known BGCs (yellow) (85). The two clusters related through a network to the viridicatumtoxin BGC represent the asperthecin BGC (bottom) and the emericellin BGC (right). The network has been manually edited to reflect curation incorporated from Inglis et al. (13). The weight of lines between clusters indicates the relatedness of the clusters as measured by the Jacard index.

We found that isolates of A. nidulans vary in total count of BGCs identified by antiSMASH, version 5.0 (33), and by manual curation. Genomes of clinical isolates MO80069 and SP260548 contain 75 and 74 BGCs, respectively, more than the 72 BGCs that we found in the A4 reference genome. Of the 72 BGCs in the reference genome, 67 were well supported by previous annotation (13), and 6 were not previously identified (see Table S1; all supplemental material is available at https://doi.org/10.6084/m9.figshare.11973717). Across all three isolates, 69 BGCs were shared (Fig. 1). Austinol synthesis requires two BGCs (34), and both were missing in SP260548 but were present in MO80069 and A4. The AN7084 BGC containing a polyketide synthase (PKS) was not observed in either clinical isolate (Fig. 1). Six BGCs were novel relative to A4, with three being present in both clinical isolates and three being unique to a single clinical isolate (detailed below). We found four BGCs in SP260548 that appeared to result from duplication of known BGCs. However, at the duplicated sites, mapping quality was poor, and read depth was half that of the surrounding genome, indicating that these duplications represented mis-assembly rather than actual genetic features. We thus eliminated one of each of these artificially duplicated clusters from further analysis.

### BGCs previously undescribed in A. nidulans.

**FIG 2:**
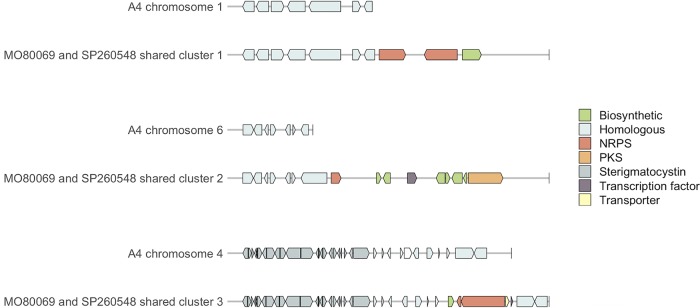
Depiction of biosynthetic gene clusters (BGCs) that were found in the genomes of the two clinical isolates SP260548 and MO80069 of Aspergillus nidulans but were missing from the A4 reference genome. The putative functionality of genes in each cluster is annotated based on protein domains. A small number of genes that had no protein domains and were unsupported by read-quality data were removed. Gene designations do not differentiate subclasses (e.g., NRPS is not distinguished from NRPS-like). All three BGCs were found in the subtelomeric region of different chromosomes, with vertical lines indicating the end of chromosomes. The three clusters are presented at different scales, with the leftmost gene being 1.3 kb, 2.5 kb, and 2.1 kb for shared clusters 1, 2 and 3, respectively. Shared cluster 3 is located between genes of the AN7838 cluster which is present in the A4 reference genome.

Of the six BGCs found here that were not present in the A4 reference genome, three were common between clinical isolates (referred to here as shared clusters) (Fig. 1). All three of these shared clusters occurred at the distal end of the subtelomeric region on different chromosomes. Shared clusters one and three were comprised of one or two nonribosomal peptide synthetase (NRPS) genes and a small number of additional secondary-metabolism-related genes (Fig. 2). While the antiSMASH call of shared cluster two included a PKS, several biosynthetic genes, and a transcription factor that were novel relative to A4, manual inspection revealed an additional NRPS and biosynthesis-related gene close by (Fig. 2). Shared cluster three is located between genes of the AN7838 BGC and may represent either the insertion of a new BGC into the genomes of clinical isolates or the loss of genes from a single BGC in the A4 reference genome. Because we cannot clearly differentiate these possibilities, we have treated them as distinct BGCs despite their nested arrangement. We observed slight differences between SP260548 and MO80069 in shared clusters two and three but were unable to distinguish these differences from assembly error due to localized areas of low-quality sequence associated with these differences. While both isolates contain these three clusters, given sequencing difficulties associated with the differences between clinical isolates, we are hesitant to infer that these BGCs differ between clinical isolates.

**FIG 3:**
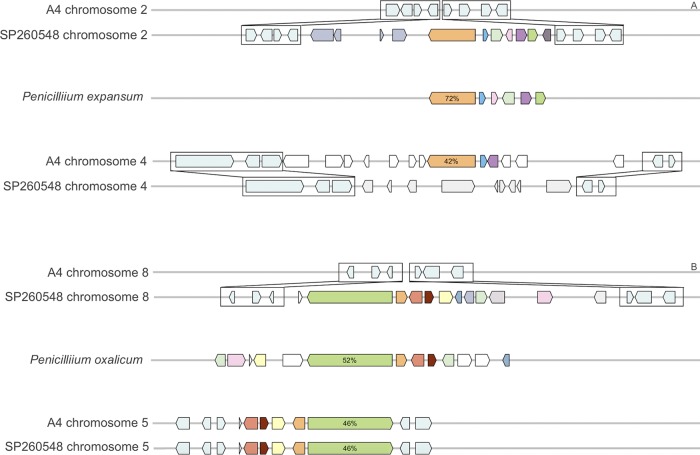
Depictions of clusters unique to SP260548. For each cluster, the corresponding locus in the reference genome A4 is indicated first, followed by the unique cluster, the most closely related cluster in *Penicillium* spp., and then the most closely related cluster in the reference that is at a different locus. While the biosynthetic gene cluster (BGC) present on chromosome 4 in A4 (AN7084 BGC) is most closely related to the first unique cluster of SP260548 located on chromosome 2, the A4 cluster has been replaced by nonbiosynthetic genes at the same locus in SP260548 (A). Conversely, the cluster on chromosome 5 of A4 (aspyridone) that is most closely related to unique cluster 2 on chromosome 8 of SP260548 is still present in SP260548 (B). Colors show genes that share identity and may be related. Percentages in backbone genes refer to protein identity with the SP260548 cluster backbone gene.

In addition to the three BGCs that were present in both clinical isolates but not A4, we also found three BGCs that were unique to a single clinical isolate. While MultiGeneBlast (35) analysis indicated that the clusters common to both clinical isolates were often closely related to existing *Aspergillus* species BGCs, the three clusters unique to a single isolate showed higher identity and synteny to known clusters in *Penicillium* spp. Given the close relatedness of *Aspergillus* and *Penicillium* genera, it is difficult to establish if such patterns of the relatedness indicate horizontal gene transfer (HGT) or the retention of the BGC from a common ancestor. A PKS cluster in SP260548 had higher identity to Penicillium expansum (73% average protein identity) and is in a different physical location than a related cluster (cluster AN7084) present in A4 (42% average protein identity) (Fig. 3A). The AN7084 cluster has been deleted in SP260548 and replaced with several genes, none of which have predicted biosynthetic functionality (Fig. 3A). Two of the genes that are located upstream of this novel cluster in SP260548 were found to contain retrotransposon-associated protein domains. BLAST of the larger of these retrotransposon-associated genes did not clarify the ancestry of this cluster as no other species had strong hits across the entirety of the protein although we did find four similar genes in the A4 genome (*AN5242*, *AN2671*, *AN2670*, and *AN0376*).

The second BGC unique to SP260548 was found to contains a PKS-NRPS hybrid backbone gene that is also flanked on one side by a gene with retrotransposon-associated protein domains. While protein identity levels of genes in this novel cluster (termed the aspyridone-like BGC) were similar between the canonical aspyridone BGC in A4 and a cluster found in Penicillium oxalicum, the related P. oxalicum cluster includes several more genes that are present in SP260548. The canonical aspyridone BGC is still present in SP260548 (Fig. 3B). The corresponding genes in P. oxalicum, however, are not completely syntenic with the aspyridone-like SP260548 BGC, indicating some divergence between these BGCs. We speculate that such patterns could be explained by divergence of these species rather than by HGT.

**FIG 4:**
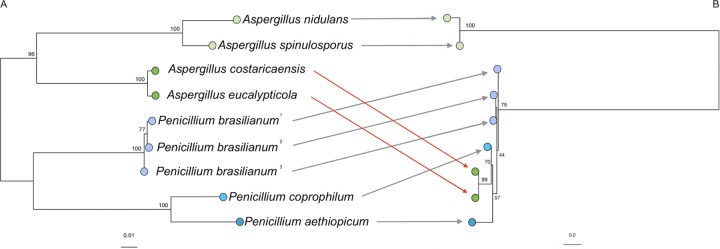
Maximum likelihood phylogenies of *Aspergillus* spp. and *Penicillium* spp. obtained from the alignment of DNA sequences from housekeeping genes *benA* and *caM* (A) and 10 genes within the viridicatumtoxin biosynthetic gene cluster (BGC) (B). Branch tips indicate the taxonomic sections of *Aspergillus* species (shades of green, from top to bottom, indicate sections *Nidulantes* and *Nigri*) and *Penicillium* species (shades of blue, from top to bottom, indicate sections *Lanata-divaricata*, *Robsamsonia*, and *Chrysogena*). Incongruent phylogenetic relationships (indicated with red arrows) are consistent with horizontal gene transfer. Aspergillus nidulans refers to the MO80069 isolates used in this study; the viridicatumtoxin BGC was not found in other isolates. Accession numbers of the genomes used are available in Table S2 (available at https://doi.org/10.6084/m9.figshare.11973717).

The third unique BGC was found in the subtelomeric region of MO80069. Similar to the novel clusters that were found in both clinical isolates in the subtelomeric region, this BGC was not associated with a retrotransposon. All 13 genes in this cluster share nearly complete synteny and orientation with the viridicatumtoxin BGC from Penicillium brasilianum, with an average protein identity of 71.4% (Fig. 4B). Given the similarity of this BGC between these two genera, we investigated the possibility that these clusters shared more recent common ancestry than could be explained by the divergence of these genera (i.e., HGT). We identified three additional *Aspergillus* spp. and two additional *Penicillium* spp. whose genomes contain this cluster. Because phylogenetic methods to detect incongruence are considered stronger than classical methods of codon usage and GC content (36), we looked for phylogenetic incompatibility by comparing DNA sequences from 10 genes in the cluster to those of 2 housekeeping genes, a method similar to previous work (37). We found that genes within the viridicatumtoxin BGC indicate closer relatedness between *Aspergillus* species in section *Nigri* and *Penicillium* species than between *Aspergillus* species from sections *Nigri* and *Nidulantes* (Fig. 4). This pattern was incompatible with phylogenetic relationships obtained from housekeeping genes for which a well-supported separation between the two genera was evident (Fig. 4). To explain how the BGC from section *Nigri* could be more closely related to *Penicillium* species BGCs than to BGCs found in section *Nidulantes*, we suggest that a common ancestor of sections *Nigri* and *Nidulantes* had the viridicatumtoxin BGC and that after these two sections diverged the viridicatumtoxin cluster from the *Nigri* lineage was horizontally transferred into the *Penicillium* genus.

### Small deletion and SNP variations in BGCs.

In addition to variation in overall BGC content, we examined the role of single nucleotide polymorphisms (SNPs) in affecting protein domains of the genes comprising BGCs. We focused our analysis on SNPs that were annotated as high impact (these mutations cause the insertion/deletion of a stop codon, frameshift mutations, etc.) by snpEFF (38). We found 12 high-impact SNPs affecting genes in BGCs, four of which were present in both MO80069 and SP260548. Most of these SNPs did not result in changes to protein domains. However, in the AN12331 BGC of MO80069, a mutation in the backbone gene removed a PKS ketoreductase domain from the resulting protein, and an SNP in *AN5363* resulted in a loss of a protein domain with unknown function. Only two of the four SNPs present in both clinical isolates resulted in domain changes, both occurring in genes of the AN3273 BGC. The first affects a putative omega-hydroxypalmitate O-feruloyl transferase in the AN3278 protein (although a transferase is still detected in both clinical isolates, with SP260548 retaining most of the original domain). The second mutation in this BGC adds an *S*-adenosylmethionine binding site to a PKS backbone protein. As loss-of-function mutations are far more common than gain-of-function mutations, the addition of a new binding site to a protein may suggest that the A4 reference genome has lost some functionality in this protein. Our results suggest that differences in the protein structure of genes in BGCs are relatively rare. While similarity in protein domains may reflect conservation of the secondary metabolome in A. nidulans, it is also a result of the small sample size used here. However, the extensive curation of genes within BGCs that we have incorporated from Inglis et al. (13) increases the likelihood that the genes included in our analysis actually function in the production of SMs and may impact the resulting SM profiles.

### Variation in SM production.

**FIG 5:**
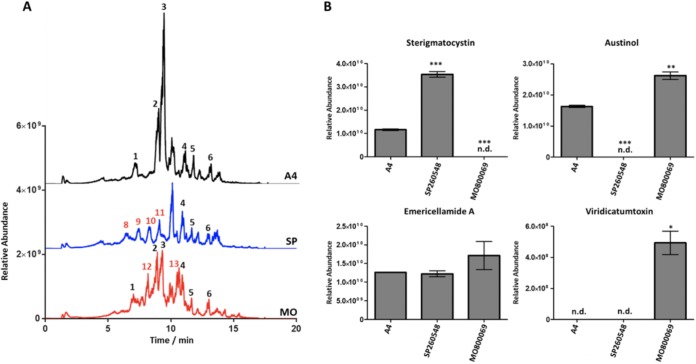
(A) A total ion chromatograms (ES− mode, linked axis) of Aspergillus nidulans isolates A4, SP260548, and MO80069. Known metabolites are annotated as follows: 1, neoaustinone/austinolide; 2, austinol; 3, dehydroaustinol; 4, emericellamide C/D; 5, emericellamide A; 6, emericellamide E/F; 8, C_15_H_26_O_4_ (269.1758); 9, C_20_H_16_O_8_ (383.0769); 10, C_20_H_20_O_8_ (387.1084); 11, C_20_H_18_O_8_ (385.0927); 12, C_28_H_32_O_12_ (559.1819); 13, C_27_H_32_O_9_/C_28_H_28_O_5_N_4_ (499.1973). Red numbers above peaks indicate that the corresponding metabolite is unique to a single isolate. (B) The relative abundances of four known secondary metabolites produced by A. nidulans isolates were determined from the mean peak area of three biological replicates. Error bars represent *±*1 standard deviation. *P* values were calculated for results from the clinical isolates in comparison to those for the A4 isolate (*, *P* < 0.05; **, *P* < 0.01; ***, *P* < 0.001; n.d., not detected).

In order to better understand how differences in BGC content between isolates of A. nidulans translates into chemotypic diversity, we assessed the production of secondary metabolites by these isolates when grown on glucose minimal medium (GMM). We found that the ultra-high-performance liquid chromatography with high-resolution mass spectrometer (UHPLC-HRMS) spectra resulting from crude extracts of the isolates used here showed a distinct secondary metabolite profiles (Fig. 5). The SM profiles for A4 and MO80069 were more similar to each other than to the profile of SP260548 (Fig. 5A; see Table S3 at https://doi.org/10.6084/m9.figshare.11973717). As predicted by bioinformatics analysis (Fig. 1), austinol and dehydroaustinol are produced by A4 and MO80069 but not by SP260548 (Fig. 5B; see also Fig. S1). We confirmed MO80069’s production of viridicatumtoxin (Fig. 4B and 5B) by comparing LC-HRMS data from this isolate to data obtained from a crude extract of Penicillium aethiopicum, a species that is known to produce viridicatumtoxin (39).

We looked in more detail at the metabolites that were produced by MO80069 and SP260548 but not A4. In addition to viridicatumtoxin, MO80069 also produced two unique metabolites corresponding to peaks 12 and 13 (Fig. 5A). SP260548 produced four unique metabolites corresponding to peaks 8, 9, 10, and 11 (Fig. 5A). HRMS data did not elucidate chemical origins of the two peaks from MO80069 but did suggest that the four SP260548 peaks could be produced by PKSs. Peaks 10 and 11 (Fig. 5A) have masses that correspond to either 5,6-dimethoxydihydrosterigmatocystin and 5,6-dimethoxysterigmatocystin, respectively, which are produced by Aspergillus multicolor (40), or the isomers aspergilol I and SC3-22-3, respectively, which are produced by Aspergillus versicolor (41). Peaks 8 and 9 (Fig. 5A) did not match any known metabolite. Despite several attempts, we were unable to isolate chemically pure versions of these metabolites to differentiate isomers.

Analysis of known compounds indicated that sterigmatocystin (ST) is not produced by the MO80069 isolate (Fig. 5B; see also Fig. S2 at the URL mentioned above). We found no deletions in the ST BGC of MO80069, and all but 15 SNPs in this region of the MO80069 genome were also found in SP260548. Of these 15 SNPs, 5 were in exons, and only 1 impacted a protein domain. The impacted gene (*AN12090* or *stcO*) is associated with converting averufin to hydroxyversicolorone (42); however, we did not find an accumulation of the averufin in this isolate. We also found no mutations in the TCG(N_5_)CGA motifs that define the binding site of AflR, which is the ST-specific transcription factor. These motifs are found upstream of several genes in the ST BGC and are required for expression of ST biosynthetic genes (43).

## DISCUSSION

We found that three isolates of A. nidulans vary in both BGC content and SM production. This diversity arises from different types of mutations, including small deletions, entire cluster loss, and SNPs. Six of the BGCs we present here were previously undescribed in A. nidulans and are related to various *Penicillium* species clusters. While the close genetic relationship between these two genera make it difficult to disentangle evolutionary relationships for several of these BGCs, we present compelling evidence that the viridicatumtoxin BGC has more recent common ancestry with *Penicillium* spp. than can be explained by the divergence of this genus from *Aspergillus*. Further, we observed the production of viridicatumtoxin by MO80069. While we found only small differences in the ST BGC of the three isolates examined here, clinical isolate MO80069 does not produce ST or any ST intermediaries, a finding which could not be explained by SNPs in this region. Together, our results demonstrate that there is considerable variation in BGC and SM production of A. nidulans. The differences in BGC content and variation in SM production we find within just three isolates of A. nidulans emphasize a need to determine if past and future inferences about secondary metabolism are applicable across the diversity of a species.

Our finding that the viridicatumtoxin BGC of *Aspergillus* section *Nigri* is more closely related to those of *Penicillium* spp. than to those found in *Aspergillus* section *Nidulantes* is compelling evidence of HGT. Recent phylogenetic assessments using whole-genome sequences suggest that sections *Nigri* and *Nidulantes* diverged between ∼52 to 70 million years ago (MYA) (44). For this cluster to appear in the three *Penicillium* sections observed here (*Lanata-divaricata*, *Robsamsonia*, and *Chrysogena*), HGT would have had to occur before the divergence of these *Penicillium* sections (estimated at ∼61 to 84 MYA [44]). The overlap of these estimated ranges (∼70 to 61 MYA) is thus consistent with our suggestion that the viridicatumtoxin BGC underwent HGT from the *Aspergillus* section *Nigri* lineage into *Penicillium* spp. sometime after this section diverged from *Aspergillus* section *Nidulantes*. While the viridicatumtoxin BGC does not appear to have been transferred directly from A. nidulans to *Penicillium* spp., our finding of a BGC in A. nidulans that has undergone HGT in a closely related lineage offers the opportunity to compare regulation of secondary metabolism in this model species to the regulation in recipient *Penicillium* spp. Many efforts to understand the expression of experimentally transferred BGCs have focused on A. nidulans (e.g., a heterologous expression system and fungal artificial chromosomes). However, questions remain about why a BGC may produce SMs in some species and not in others (45, 46). Recently de Reus et al. (47) demonstrated that artificially transferring the aurofusarin BGC from *Fusarium* spp. into A. nidulans removed nitrogen-related regulation of the cluster that was evident in the donor species. Estimates from natural isolates suggest that 0.1 to 5% of genes in fungi, and perhaps more in Pezizomycotina (a group containing *Aspergillus*) (48), are thought to be the result of HGT (49, 50). Indeed, HGT of secondary-metabolism BGCs may occur or be retained more often than other types of gene clusters (19). Experimental transfer of BGCs may result in unintended compensatory mutations and off-target effects (51, 52). We suggest that the natural transfer of this BGC from a close relative of A. nidulans into *Penicillium* spp. offers the opportunity for future work to examine the role of HGT in SM regulation to achieve insights for both basic and applied research.

While A. fumigatus is the most prevalent pathogen in the *Aspergillus* genus, A. nidulans infections may be more resistant to antifungals and may result in higher mortality in some patients (53). Bastos et al. (25) recently found that MO80069 has significantly higher virulence in neutropenic animal models than SP260548 and the A4 reference isolate. In A. fumigatus, strain-specific differences in virulence levels are well known (54), but questions have remained about what the underlying causes of these differences are. We speculate that some of the differences in secondary metabolism we observed here could contribute to the strain-specific virulence observed in MO80069. Viridicatumtoxin is a rare example of a fungal compound built upon a structure similar to tetracycline antibiotics (39) and can inhibit the growth of antibiotic-resistant Staphylococcus aureus at higher activity than even tetracycline (55). However, this compound derives its “toxin” designation from nephrotoxicity observed in rats (56). While the finding of this toxin in the more virulent MO80069 raises questions about previously observed differences in virulence, Knowles et al. (57) recently found known SM virulence factors in nonpathogenic fungi, thus raising the point that virulence is not always caused by a single trait but can also arise out of the sum or interaction of several traits. Our finding of diversity in secondary metabolism of A. nidulans presents the opportunity to better understand how the presence of specific BGCs or the interactions between BGCs may affect fungal virulence.

In addition to viridicatumtoxin, we discovered five BGCs that were previously unknown in A. nidulans. Previous assessments of intraspecific variation in A. fumigatus BGCs by Lind et al. (24) found that some isolates were missing or had pseudogenized variants of up to 30% of BGCs common in the species. Such findings raise concerns about the possibility that commonly used reference isolates may be aberrant in BGC content relative to that of the species they represent. Of the 78 total BGCs we found across the three isolates assessed here, 69 were present in all three isolates, and only 6 were new relative to A4 content. While sampling of more isolates will likely reduce the portion of BGCs present in both A. nidulans isolates and in A4, our results do suggest that the A4 reference genome is not particularly aberrant in the number or content of BGCs. While this result is encouraging for the applicability of past work to this species, more sampling is needed to further clarify the frequency of clusters identified here.

The diversity we find both in BGC content and in SM production gives some context to previous work on the regulation of secondary metabolism in fungi. The production of ST is thought to be correlated with the production of various fungal structures in *Aspergillus* spp. (58), particularly asexual sporulation (26). Anecdotally, we note that SP260548, which did not produce ST under conditions used here, has very low production of sexual spores; conidial production, which is also associated with a loss of ST (26), was not measured (25). In A. flavus the production of aflatoxin (sterigmatocystin is the immediate precursor to aflatoxin) is also thought to be positively associated with conidial production (59–62). However, a search for such associations in natural isolate studies often has conflicting results (63, 64; M. T. Drott, T. R. Satterlee, J. M. Skerker, B. T. Pfannenstiel, N. L. Glass, N. P. Keller, and M. G. Milgroom, submitted for publication). Such discrepancies highlight that given the diversity present in fungal species, associations evident in the lab using a single reference isolate may not be informative of natural patterns. The diversity we observe here in the secondary metabolism of A. nidulans presents the opportunity to better expand our understanding of associations between secondary metabolism and primary metabolism to better reflect the genetic diversity of species.

Several papers have recently focused on how chemotypic diversity within fungal species may be harnessed for drug development (14, 15). While there has been some evidence of intraspecific BGC diversity on the genomic level (24, 46), there has been little work that ties this diversity to SM production. Our results show that there is considerable variation in both BGCs and in subsequent SM production even in what is perhaps the most well-studied SM model, A. nidulans. We speculate that given the development of heterologous expression (65) and fungal artificial chromosome (66) systems in this species, novel metabolites discovered within this species may be more readily accessible. In addition to applied implications of our work, we suggest that the natural variation in secondary metabolism uncovered here in A. nidulans offers avenues to study how differences in BGCs and evolutionary history impact the regulation of and associations with SM production.

## MATERIALS AND METHODS

### BGC identification and assessment.

We identified secondary-metabolite BGCs from two clinical isolates and the A4 reference isolate of A. nidulans that were previously sequenced using both Nanopore long reads (Oxford Nanopore Technologies, Oxford, United Kingdom) and Illumina short reads (Illumina, San Diego, CA) (25). Genes were annotated using Augustus, version 3.3.2 (67), with default gene models for A. nidulans. Genomes of all isolates were scanned for BGCs using antiSMASH, version 5.0 (33). Because borders of BGCs called by antiSMASH are sometimes inaccurate, we amended both the total count of BGCs and the borders of BGCs using a curated list developed by Inglis et al. (13). We annotated the potential impact of SNPs and indels that were found in the genes comprising BGCs with SnpEff, version 4.3T (38), using existing variant data (25). SNPs and indels that affected stop codons or caused frameshifts were annotated as high impact. We established if high-impact changes affected protein domains of genes in BGCs using the NCBI conserved domains. Additionally, we looked for mutations to AflR binding motifs TCG(N_5_)CGA (43) found within the sterigmatocystin BGC.

We determined which BGCs differed by presence/absence from the A4 reference by creating BGC networks with BiG-SCAPE (68) and visualizing these networks with Cytoscape, version 3.7.1 (69). Additionally, we assessed which genes were missing within BGCs using reciprocal best-hit BLAST analysis implemented for all genes using a custom script (24). We confirmed the deletion of genes and entire BGCs by creating alignments with BWA-MEM, version 0.7.17 (70), and summarizing quality data using bedtools, version 2.28.0 (71). When necessary, alignment data were manually inspected using the Integrative Genomics Viewer (72). Novel BGCs were confirmed relative to the A4 reference genome using both blastn and blastp from the BLAST+ suite, version 2.8.1 (73). When a BGC was confirmed as novel relative to the A4 reference genome, we looked for related clusters in other species using MultiGeneBlast, version 1.1.14 (35). Protein identity comparisons were conducted using a global alignment implemented in EMBOSS, version 6.6.0 (74). This method identified the viridicatumtoxin BGC (see Results) as having high protein identity and synteny with species outside *Aspergillus* spp., patterns that are consistent with HGT. In order to confirm this HGT, we identified the presence of this BGC in other species by querying the GenBank assembly and protein Eurotiales database entries as well as the RefSeq nonredundant databases (downloaded on 1 February 2020). Nucleotide sequences that fell within 50,000 bp and proteins whose accession numbers were within 30 of each other were considered clustered. We selected species in which we confirmed that at least 11 of the 13 genes found in the A. nidulans viridicatumtoxin BGC were clustered. When identification of the taxonomic section of selected species was not present in Steenwyk et al. (44), we referenced Houbraken et al. (75) and Varga et al. (76) to obtain this information. We used SAMtools, version 1.9 (77), to obtain the sequence of homologous regions from BLAST hits for two distinct sets of genes: a set of 10 genes from the viridicatumtoxin gene cluster and a set comprised of the housekeeping genes *benA* and *caM*. Each set of genes was concatenated and aligned, and gaps were removed using MEGA, version 10.0.x (78), for a final length of 13,948 bp and 540 bp, respectively. We constructed a maximum likelihood tree for each gene set using MEGA, version 10.0.x (78). Phylogenetic trees and depictions of clusters were drawn using the R packages tidyverse (79) and gggenes (80).

### Fungal extraction and SM analysis by UHPLC-HRMS.

To assess the production of secondary metabolites, we grew the three isolates on glucose minimal medium (GMM) at 37°C for 14 days and performed extractions with ethyl acetate, similar to a previous methodology (81). The organic phase was washed with water, dried over anhydrous magnesium sulfate, and concentrated under reduced pressure. Resulting crude extracts were resuspended in LC-MS-grade acetonitrile (10 mg/ml) and filtered through an Acrodisc syringe filter with a nylon membrane (Pall Corporation, New York, NY) (0.45-μm pore size).

To identify and quantify metabolites from crude extracts, we performed UHPLC-HRMS on a Thermo Fisher Scientific-Vanquish UHPLC system (Thermo Fisher Scientific, Waltham, MA) connected to a Thermo Fisher Scientific Q Exactive Orbitrap mass spectrometer in electrospray-positive (ES+) and ES-negative (ES−) modes between 200 *m/z* and 1,000 *m/z* to identify metabolites. A Zorbax Eclipse XDB-C_18_ column (2.1 by 150 mm; 1.8-μm particle size) (Agilent Technologies, Santa Clara, CA) was used with a flow rate of 0.2 ml/min for all samples. LC-MS-grade water with 0.5% formic acid (solvent A) and LC-MS-grade acetonitrile with 0.5% formic acid (solvent B) were used with the following gradient: 0 min, 20% solvent B; 2 min, 20% solvent B; 15 min, 95% solvent B; 20 min, 95% solvent B; 20 min, 20% solvent B; 25 min, solvent B. Nitrogen was used as the sheath gas. Data acquisition and processing for UHPLC-HRMS were controlled using Xcalibur software (Thermo Fisher Scientific). Files were converted to the mzXML format using MassMatrix MS Data File Conversion (82) and analyzed in MAVEN (83) and XCMS (84). We compared the mean peak areas of different compounds using an unpaired Student’s *t* test in GraphPad Prism, version 6.0 (GraphPad Software, La Jolla, CA).

### Data availability.

Short-read sequences for these strains are available in the NCBI Sequence Read Archive (SRA) under accession numbers SRR10983230, SRR10983231, SRR10983232, and SRR10983233 and BioProject number PRJNA603646. Genomes were deposited in GenBank under accession numbers JAAFYM000000000 and JAAFYL000000000. Curation of secondary metabolite clusters present in the A4 reference genome as well as HRMS spectra for a select subset of compounds is available in the supplemental material (available at https://doi.org/10.6084/m9.figshare.11973717).
